# Large-Scale Assessment of a Fully Automatic Co-Adaptive Motor Imagery-Based Brain Computer Interface

**DOI:** 10.1371/journal.pone.0148886

**Published:** 2016-02-18

**Authors:** Laura Acqualagna, Loic Botrel, Carmen Vidaurre, Andrea Kübler, Benjamin Blankertz

**Affiliations:** 1 Neurotechnology Group, Technische Universität Berlin, Berlin, Germany; 2 Institute of Psychology, University of Würzburg, Würzburg, Germany; 3 Bernstein Focus Neurotechnology, Berlin, Germany; 4 Machine Learning Group, Technische Universität Berlin, Berlin, Germany; University of Electronic Science and Technology of China, CHINA

## Abstract

In the last years Brain Computer Interface (BCI) technology has benefited from the development of sophisticated machine leaning methods that let the user operate the BCI after a few trials of calibration. One remarkable example is the recent development of co-adaptive techniques that proved to extend the use of BCIs also to people not able to achieve successful control with the standard BCI procedure. Especially for BCIs based on the modulation of the Sensorimotor Rhythm (SMR) these improvements are essential, since a not negligible percentage of users is unable to operate SMR-BCIs efficiently. In this study we evaluated for the first time a fully automatic co-adaptive BCI system on a large scale. A pool of 168 participants naive to BCIs operated the co-adaptive SMR-BCI in one single session. Different psychological interventions were performed prior the BCI session in order to investigate how motor coordination training and relaxation could influence BCI performance. A neurophysiological indicator based on the Power Spectral Density (PSD) was extracted by the recording of few minutes of resting state brain activity and tested as predictor of BCI performances. Results show that high accuracies in operating the BCI could be reached by the majority of the participants before the end of the session. BCI performances could be significantly predicted by the neurophysiological indicator, consolidating the validity of the model previously developed. Anyway, we still found about 22% of users with performance significantly lower than the threshold of efficient BCI control at the end of the session. Being the inter-subject variability still the major problem of BCI technology, we pointed out crucial issues for those who did not achieve sufficient control. Finally, we propose valid developments to move a step forward to the applicability of the promising co-adaptive methods.

## Introduction

Brain Computer Interfaces (BCI) are devices that let the users control a technical device or a computer application just by using the modulation of the neural activity, without explicit muscular output [1–3]. One of the main applications of this technology is the clinical use for people affected by a severe degree of paralysis, who are not able to move, speak or reliably control the movements of their eyes [4–10]. In this context, a widely investigated type of BCI is that based on the modulation of the Sensorimotor Rhythm (SMR) as measured by the electroencephalogram (EEG) [11, 12]. The performances and usability of SMR-based BCIs have improved in the last decades: long training sessions, in which the users had to learn to control specific brain features, have been replaced by the machine-learning based approach that allows for an effective performance from the first session [13, 14]. Then, further improvements have been made to compensate for the non-stationarity of the EEG signal, in particular when transitioning from calibration runs to evaluation runs. Several recent studies proposed adaptive learning for EEG signals, either based on the update of the feature space [15–18] or of the classifier [19–22]. The bottleneck of SMR-based BCIs is that a significant percent of users (estimated between 20–50% [23, 24]) is not able to modulate the SMR in a volitional way (BCI inefficiency) [25, 26]. The causes of this phenomenon are unclear and vary from person to person. For example, it has been observed that for some participants the classifier cannot be successfully trained, meaning that no difference in the modulation of the SMR can be found between motor imagery tasks. In the classical case, which relies on offline calibration data, the BCI inefficiency may be due to the changes of the brain features between the offline training session and the online feedback. In this specific scenario the development of co-adaptive BCI system has been crucial, which lets the people interact with the feedback from the first trials, removing the transition from offline to online phase. A promising approach is described in two studies by Vidaurre and colleagues [21, 26], who used adaptive machine learning methods to eliminate offline calibration. The participants were selected for their categorization of BCI control after participating in previous studies. For categorization we refer to that of Vidaurre et al. [27]: for Category I users (Cat I), the classifier can be successfully trained and they gain good BCI control in the online feedback session. For Category II users (Cat II) the classifier can be successfully trained but no good performances can be achieved in the feedback phase. For Category III users (Cat III), no successful training of the classifier is achieved. The co-adaptation scheme presented in [21, 26] started with a pre-trained subject-independent classifier operating on simple features, with which the user interacted continuously from the first run. Then, a subject-optimized classifier was used along with the supervised co-adaptation, for three runs. In the last runs, an unsupervised adaptation scheme was adopted to track the drift of the features during the session. This novel approach let the users who did not show BCI control with the classical machine learning approach gain BCI control within one session. The BCI users who were already able to effectively operate the BCI gained accurate control within few minutes.

It is important to develop advanced methods to solve the BCI inefficiency, but also it is useful to identify a priori the potential users that may have difficulties to adopt one particular approach. It would allow to skip a frustrating experience and to assign them to a different BCI system (e.g., ERP based). Growing interest in this topic led to investigate neurophysiological predictors of BCI performances [25, 28–32]. For example, Blankertz and colleagues [25] determined a neurophysiological predictor from a two minutes recording in condition ‘relax with eyes open’, using two Laplacian EEG channels over the right and left motor cortex. The predictor was based on the power spectral density (PSD) of the alpha and beta bands. They obtained a correlation of r = 0.53 (r = 0.61 after outlier rejection) between the proposed predictor and BCI feedback performance of a database of 80 BCI-naive participants operating an online SMR-BCI. A different approach is described by Suk et al. [29] in which they investigated a novel probabilistic framework on the same dataset. The novel method was used to predict the BCI performance using the 2 min resting-state EEG data and 3 Laplacian channels (C3, Cz, C4). After clustering the participants based on their spectral feature vector, they built a linear regression model based on the grouping. They could predict the participants performances on the later BCI session with a maximum correlation coefficient of 0.581. A more recent study by Zhang and colleagues [31] derives the SMR-BCI predictor from resting-state EEG recording calculating the spectral entropy at different channels. They found that the entropy at channel C3 for the eyes closed resting-state condition had the high correlation of 0.65 with offline SMR-BCI performance. They also extended the evaluation of the spectral entropy predictor to inter-session prediction, achieving classification accuracy up to 89%. The same group further investigated offline SMR-BCI performance variations through the analysis of EEG resting-state networks, resulting in a reliable prediction [32]. Other studies extended the investigation of the correlation between different neurophysiological predictors and BCI performance also on single-trial bases, [33–35].

These studies that address the problem of BCI inefficiency, from one side developing new algorithms to cope with inefficient BCI control and from the other side to predict the success of the BCI, have been conducted with a relatively low number of participants. In particular, it has not been assessed yet whether an adaptive system using the state of the art machine learning technology can be successfully applied in large scale, without the direct intervention of BCI experts on sensible parameters. In order to tackle this point, a large-scale study has been conducted in two different locations, the Technical University of Berlin and University of Würzburg. From the BCI perspective, the two main goals of the study were: first, to achieve the successful results of the previous studies based on co-adaptive calibration in naive un-categorized users and with a fully automatic system. Second, to test the neurophysiological predictor in a new larger pool of participants. Therefore, a sample of 168 participants was recruited to conduct a co-adaptive EEG-based SMR-BCI, in single-session study. The BCI session immediately started with online feedback, applying the co-adaptive techniquesmethods described in [21, 26]. However, differently from [21, 26], the proposed system was fully automatized, i.e. the experimenters did not manipulate the selection of the features or the parameters of the classifier or the adaptation scheme. This approach was meant to simulate a real case in which an adaptive system would be in-home applied by users not familiar with the sophisticated machine learning algorithms. Nevertheless, since the participants were totally naive in respect of BCI technology, it was necessary that BCI experts provided them with the instructions about how to correctly perform motor imagery. The brain resting state activity of all participants was acquired prior to the BCI task and allowed a later offline investigation of the neurophysiological predictor of BCI performances, according to the methods described in [25]. Moreover, two different training strategies were adopted which correspond to the psychological factors of degree of concentration and visuo-motor coordination that were found in [36] to correlate with BCI performance. In the current study, these training strategies were used as interventions prior to the BCI session in order to assess their ability to improve BCI performance. The results are discussed taking into consideration the different training groups, but a more detailed investigation of the effect of the interventions will be published elsewhere (manuscript in preparation).

## Methods

### Participants

Hundred-sixty-eight (168) participants were recruited for the study. 83 experiments were conducted at the Technical University of Berlin and 85 at the University of Würzburg. Participants were all naive in respect to BCIs. Eight participants were excluded from the analysis for taking Central Nervous System (CNS)-affecting medication or reporting a psychiatric disorder, e.g. ADHD or depression. Four participants were excluded because of technical problems in the EEG setup and 5 for not complying with the instructions given. So, the final sample was reduced to 151 participants (96 females, mean age 24.9, SD = 6.5). The study was conducted in accordance with the declaration of Helsinki, approved by the Ethical Review Board of the Medical Faculty of the University of Tübingen, and written informed consent was obtain prior to the experiment. Participants received 8 Euro per hour for their participation.

### Apparatus

EEG was recorded with the sampling frequency of 1000 Hz using BrainAmp amplifiers and ActiCap active electrode system with 64 channels (both from Brain Products, Munich, Germany). The electrodes used were: Fp1,2, AF3,4, F1-10, Fz, FC1-6, FCz, FT7,8, CFC1-6, T7,8, C1-6, Cz, CCP1-6, CP1-6, CPz, TP7,8, P1-6, Pz, PO3,4, O1,2, A2. All the electrodes were referenced to the left mastoid, grounded to the forehead. For offline analyses, electrodes were re-referenced to linked mastoids. All impedances were kept below 10 kOhm. The stimuli were shown on a 23” screen with a native resolution of 1920x1080 pixels at a refresh rate of 60Hz. Participants sat on a comfortable chair at a distance of approximately 80 cm form the display.

### Design and procedure

#### Interventions and pre-measurments

Participants were randomly assigned to one of three groups, in which different kinds of intervention were performed before the BCI session. The aim of the interventions was to investigate whether different pre-training could predict or even enhance SMR-BCI performances. The first group performed a progressive muscular relaxation (PMR), following the instructions of a Jacobson Progressive Muscle relaxation audio recording of 23 minutes. The second group practiced a two-hand visuo-motor coordination task (inspired by the 2HAND test (Schuhfried GmbH), in which they had to steer a ball along virtual paths, controlling with the right hand the knob that set the horizontal position and with the left hand the knob of the vertical position [36]. In Würzburg this task was performed for 5 paths of increasing difficulties, with an average duration of 7.6 min (SD = 2.6). In Berlin, the paths were cycled for a total duration of 23 min. The third group was the control group, and participants had to spend 23 min reading a text about BCI technology and answer to related questions. After the intervention, participants started the BCI session. The first measurement consisted in recording the brain activity during resting state. A recorded voice instructed the participants to close the eyes for 15 s and open the eyes for an other 15 s. While with open eyes, they had to look at a geometrical moving shape at the middle of the display. The cycle ‘eyes open-closed’ was repeated for 10 times. The SMR BCI motor imagery (MI) part followed the resting state measurement. The total duration of the experiment was approximately 3.5–4 hours, including the preparation of the EEG cap (about 1 hour), intervention and EEG recording (about 1.5 hours), pauses and hair wash in loco.

#### Design of the BCI feedback

The BCI feedback used in the study consisted of a cursor with cross shape, which was displayed in the center of the screen for 2 seconds, followed by a white arrow-shaped cue, those direction indicated the MI to perform: right direction for MI of the right hand, left direction for MI of the left hand and bottom direction for MI of the feet. The user was instructed to perform the MI as soon as the cue was displayed and the cross turned color from black to magenta. After 1 second, the feedback was displayed for 3 seconds. In this period, the output of the classifier was translated into cursor movement in a rate control manner, i.e. every 40 ms a fraction of the classifier output was added to the current cursor position. When the 3 seconds of feedback were over, the color of the cursor turned black again and a new trial started. The final position of the cross determined the success or failure of the trial. For the first three runs, a positively biased feedback was employed. This feedback manipulation was not present in previous co-adaptive studies [21, 26] and it will be explained in the next section. Here, the cursor could only move from the center position towards the indicated target direction. The number of trials varied in the different parts of the experiment, but in all the runs after 20 trials there was a short break of 15 seconds.

#### Online motor imagery BCI

**Fig 1:**
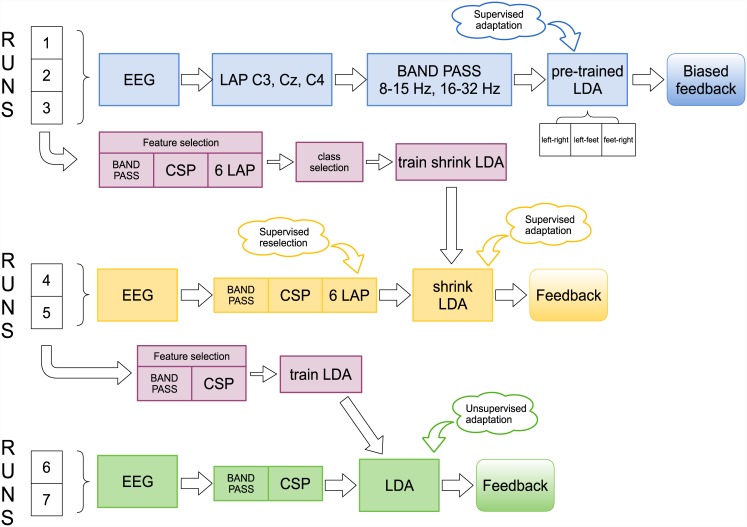
Schematic flowchart of the online protocol. The EEG processing and adaptation protocol during runs 1–3 with positive biased feedback are depicted in blue, in yellow the processing and adaptation during runs 4–5 with real feedback, in green the processing and adaptation during runs 6–7 also with real feedback. The adaptation applied in runs 1–3 and 4–5 uses supervised methods, the adaptation of runs 6–7 uses unsupervised methods. In magenta are depicted the phases of subject-specific features selection (e.g. frequency band, CSP filters, etc) and training of the classifier that happened two times, i.e. after runs 1–3 and after runs 4–5.

The motor imagery session consisted of 3 parts: the first part comprised 3 runs (1–3), of 40 trials each. The second and the third part had 2 runs (here referred as runs 4–5 and runs 6–7), of 80 trials each (Fig 1). Between the runs there was a short pause to let the participants relax. In runs 1–3, EEG was preprocessed using three Laplacian derivations [37] over C3, Cz and C4, calculated from four surrounding channels, equally weighted and subtracted from the central one. The signal was also frequency-filtered in the alpha (8–15 Hz) and beta (16–32 Hz) bands using two Butterworth filters of order 10. Three binary subject-independent classifiers were adopted, one for each pair of classes, *left-right*, *left-feet* and *feet-right*. They were trained on a dataset of 48 people whose performance was above 70% in a previous study [25]. The classifier was based on linear discriminant analyses (LDA). In this phase, the LDA classifier was adapted in a supervised manner after every trial using the Adaptive Mean Estimation and Adaptive Inverse Covariance Matrix Estimation algorithms (see [21, 26] for a detailed description). The inverse of the covariance matrix and class mean values were updated after every trial using the class label (type of motor imagery task) only for the mean values of the past trial. Feedback was provided to the participants slightly differently from the procedure previously described. In this phase, the movement of the cursor was positively biased in the following way. The two binary classifiers including the target class were evaluated. If one of them had a positive output for the target class, the cursor was moved proportionally towards the target direction. Otherwise, the cursor would slowly move back towards the center position, but it was never moved in direction of one of the two wrong classes. The use of a positively biased feedback was motivated by the fact that Cat II and III users were on average not able to reach significant good performance (over 70% of accuracy) in the initial phase [21, 26]. This lack of control could therefore irritate the user [38]. Positive feedback was already found to be useful in BCI training [39]. Data recorded in runs 1–3 were used to select subject-specific settings (according to the tutorial in [40]), which were used in runs 4–5. The frequency band in which the classes were better discriminated was selected. A second LDA classifier with shrinkage of the covariance matrix [41] was trained on log-band power features extracted from the Laplacian and Common Spatial Patterns/Filters (CSP/CSF) [42] based on 24 EEG channels. In this training, the pair of classes that showed the highest classification accuracy (using cross-validation) was chosen and used for the rest of the experiment. This classifier was applied in runs 4–5. The CSF were selected automatically for each participant using the bandpass filtered data of runs 1–3. The number of selected CSF varied between 2 and 6 and they remained fixed during runs 4–5. In order to allow some spatial adaptation, six Laplacian derivations were selected for each participant based on their discriminative power (quantified by the biserial correlation coefficient) and concatenated to the CSP channels, leading to a feature vector of dimension between 8 and 12. Two Laplacian channels were selected from the left hemisphere, 2 from the center and 2 from the right hemisphere. In runs 4–5 the adaptation was performed in supervised manner. After every trial, the selection of the six Laplacian channels was updated using the last 100 trials and the classifier recalculated. Data recorded in runs 4–5 (160 trials) was used to recalculate the frequency band and new CSP features based on 47 EEG channels, and thereafter to train the LDA. In runs 6–7 no additional Laplacian channels were used. During these last 2 runs, after each trial the linear classifier was adapted in unsupervised manner, using the adaptation of the pooled mean. The adaptation of the pooled mean produces a shift of the classifier’s hyperplane, tracking the position of the mean of the features. The value of the update coefficient used in this study was 0.05, selected according to [43]. In runs 4–5 and 6–7, the feedback was not biased and the cursor position was update according to the classifier output as described in the previous section. Thus, the final position of the cursor reflected the success or failure of the trial.

### Pre-processing and data analysis

EEG signal was lowpass filtered from 0 to 40 Hz with a Chebyshev filter of order 10 (3 dB of ripple in the passband and 40 dB of attenuation in the stopband) and down-sampled to 100 Hz. For ERD/ERS visualization, the class combination chosen in the first calibration after runs 1–3 was selected for each participant. The chosen pair of MI classes did not change during the following part of the experiment. Data were filtered using the specific band selected during this calibration. The grand average ERD/ERS was done over the participants having the same combination of selected classes for each adaptation scheme, i.e. runs 1–3, runs 4–5 and runs 6–7. For online classification performances we referred to the results of runs 4–7, in which the actual classifier output was translated into cursor feedback. The percentage of feedback accuracy refers to the number of trials in which the final position of the cursor was in line with the target class. Mean feedback accuracies over 54.69% were considered significantly higher than chance level [44]. This threshold was calculated with the binomial inverse cumulative distribution function (cdf), which returns the minimum number of trials such that the binomial cdf is equal to or exceeds 0.95. Given that the total amount of feedback trials was 320 with 0.5 probability of success, the result of the the binomial inverse cdf was 175, i.e. 54.69% of successful trials. Two-sample t-test was run between the performances of participants in Berlin and Würzburg, in order to check if the difference between the average accuracies of the two laboratories were statistically significant. The measurement of the brain in resting state was used to extract the neurophysiological predictor of BCI performances. Two Laplacian derivations, C3 and C4, were calculated from 9 monopolar channels. For each Laplacian derivation, we concatenated the time intervals in which the participants had eyes opened and divided the continuous data into 2 s epochs. The power spectral density (PSD) was calculated for the two derivations (between 2 Hz and 34 Hz, with a step of 0.5 Hz) and smoothed with a moving average filter (window 3 Hz). The PSDs were averaged over epochs. The difference between the maximum PSD of each derivation and the fit of the 1/f noise spectrum was calculated. The average of the two values determined the predictor. These values represent the estimate of the strength of the SMR rhythm over the motor areas. For a more detailed description about the modeling of the PSD and 1/f curves, please refer to [25]. Three participants were excluded in this analysis because of the bad quality of the resting state recording. Therefore, the predictor was evaluated on a pool of 148 participants. Statistical tests run between different intervention groups and between different runs were done using one way-ANOVA with multiple comparisons to test which means were significantly different. The assumption of normality distribution of the data was checked with the one-sample Kolmogorov-Smirnov test. The assumption of equal variances was tested using Bartlett’s test.

## Results

### Online performances

**Fig 2:**
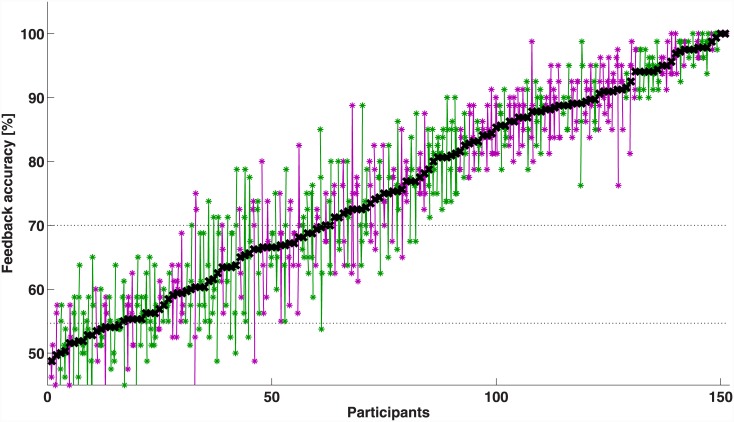
Overview of BCI performances sorted in ascendent order. For each participant, the black crosses indicate the overall mean feedback accuracy and the colored stars the accuracies of the four feedback runs (4–7). The participants of Berlin are displayed in magenta and the ones of Würzburg in green. The accuracies of each participant are connected by colored lines that emphasize the variance of the accuracies between runs. The dotted gray lines represent the accuracy considered necessary for BCI control (70%) and the threshold for accuracy significantly higher than chance (54.69%).

The following results refer to classification performances of runs 4–7, in which the feedback was representing the actual classifier output. We consider as correct trials those in which the final position of the cross was on the display at the correct side of the target class (right for right MI, left for left MI and down for feet MI) with respect to the starting point at the center of the display. The classification accuracies represent the percentage of correct accomplished trials. Fig 2 depicts the feedback accuracies for each participant, sorted in ascendant order. The black crosses represent the average accuracies of the overall feedback phase and the colored dots the accuracy of each run. The values of the participants tested at TU Berlin are depicted in magenta, those of the participants of Würzburg in green. A total of 135 participants had mean classification performance significantly better than chance. Among these, 90 had mean accuracy over the threshold considered necessary for efficient BCI control (i.e. 70% [38]), with a mean of 85.65% (SE = 0.90), and 45 participants had mean accuracy of 62.47% (SE = 0.70). The other 16 participants had mean accuracy not significantly higher than chance level, 52.19% (SE = 0.44). From the plot it is visible that many participants show variable performances between runs, especially those who have an average accuracy between 60% and 80%. Considering the whole sample of participants, the variability between runs was not significant (p = 0.67). However, the participants who performed on average at chance level had a significant increase of accuracy at run 6 compared to runs 4–5 (p<0.01), performing significantly better than chance in the last two runs. The plot also shows a different distribution between the average accuracies of the participants recruited in the two universities. Two-sample t-test run between the accuracies of the two groups showed a significant effect of the location (p<0.05), with participants of Berlin having higher mean accuracy than participants of Würzburg. Therefore, the following results are presented considering not only the different intervention groups, but also the location of the study.

**Fig 3:**
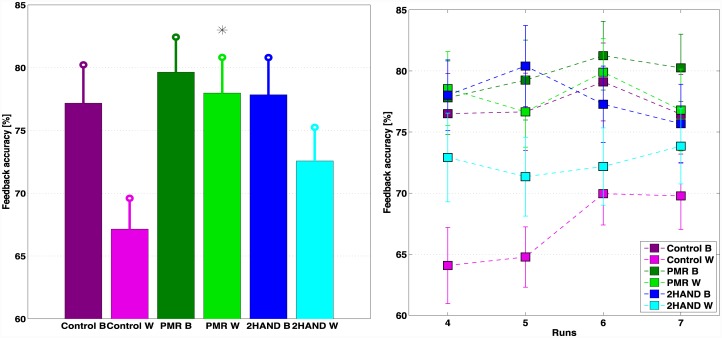
Mean BCI feedback accuracies for each intervention group (Control, PMR, 2HAND) over runs 4–7 (left) and run-wise (right). ‘B’ refers to participants of Berlin and ‘W’ to participants of Würzburg. PMR W has significantly higher mean BCI performance compared to Control W (p<0.05), marked with a star in the bar plot.

Fig 3 (left) depicts the mean feedback accuracies for the different intervention groups. The groups are labeled according to the intervention performed (Control, PMR, 2HAND) and to the location of the experiment (‘B’ for Berlin and ‘W’ for Würzburg). Participants who performed progressive muscular relaxation show the highest average BCI performances, followed by participants who did motor coordination training and control group, in the respective locations. In Würzburg, the mean performances are significantly different (p<0.05), and the difference is significant for the PMR group as compared to the control group, but not for the 2HAND group. Note that the motor coordination training was performed in Würzburg for about 7.6 min (SD = 2.6) versus the 23 minutes in Berlin. The control group of Berlin has an average accuracy higher than the control group in Würzburg, so even though the PMR group has higher performances, the difference is not significant (p = 0.82). Fig 3 (right) depicts in detail the trend of the classification accuracy run-wise for the corresponding groups. Note that after run 5 the classifier was re-trained and in the following runs a different adaptation scheme was adopted. In general, the groups that begin at run 4 with average classification higher than 70% do not show a great variation of performances between runs. Both PMR groups and Control B have the same trend, with slightly higher accuracy at run 6 after the second training of the classifier, and decreasing in the last run. Group 2HAND B instead had the opposite trend, with decreasing accuracy at run 6. Control W, which had the lowest classification performances, had a substantial increase of average accuracy after run 5, reaching the threshold of 70% in the last two runs. Note that the average final performance of all groups is at the threshold of control or higher.

### Grand Average ERD/ERS

**Fig 4:**
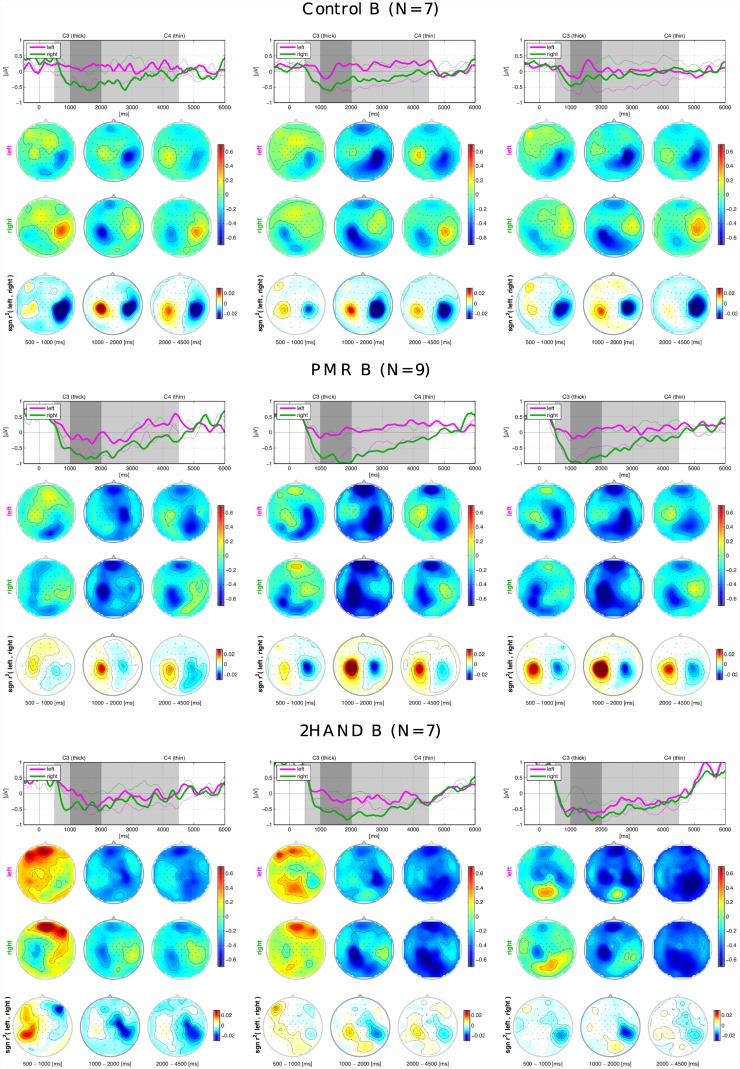
Grand average ERD/ERS for class combination. *left-right* and intervention groups in Berlin. ‘N’ is the number of participants of each group. From left to right: runs 1–3, runs 4–5, runs 6–7. The time plots in the first rows picture the evolution of the ERD/ERS for about 6000 ms at C3 (thick lines) and C4 (thin lines). At time 0 is the onset of the cue, at times 1000–4000 the display of the feedback. Magenta lines refer to *left* MI trials, green lines to *right* MI trials. The scalp plots underneath refer to the shaded areas of the time plots and show the distribution of the ERD/ERS. In the second rows, the scalp plots of the *left* MI trials, in the third rows the scalp plots of the *right* MI trials and in the fourth the scalp plots of the sign-r^2^.

Fig 4 shows the grand average ERD/ERS across participants whose chosen class combination was *left-right*, for the different intervention groups tested in Berlin and different types of adaptation used in the experiment. In each column, in the first row is depicted the time evolution of the ERD/ERS between the onset of the cue on the display indicating the class of the motor imagery to perform (0 ms) and 6000 ms after. Feedback was displayed between 1000 ms and 4000 ms. The shaded areas divide the whole time course in three areas of interest, one short interval referring to the 500 ms before the start of the feedback and the other two during the motor imagery performed in presence of feedback. The average ERD/ERS values in these three intervals are calculated for each channel and depicted as scalp plots in the second and third rows. The second row refers to trials in which left motor imagery was done, the third to right motor imagery. The fourth row pictures the values of the sign-r^2^ as a measure of discriminability between the two classes. The participants of the PMR group show more pronounced class-wise ERD as compared to the other two groups, which results in high discriminability between the two classes. The control group shows ERD topographies more localized than the PMR and visible ERS in the ispilateral hemispheres, especially during the right motor imagery. This leads to a high discrimination between classes also in this group. In both groups, the interval between 1 s and 2 s of feedback is that one in which the ERD/ERS are more pronounced. Nevertheless the modulation of the SMR in both classes is already visible 500 ms before the beginning of the feedback. The 2HAND group shows different patterns. The ERD begins mainly after the beginning of the feedback, and no clear pattern is visible in the preceding 500 ms interval. After 1 s the ERD becomes pronounced, especially in the last interval of the feedback. Even though deeper ERD is visible compared to the control group, especially in runs 1–3 and 6–7, the desynchronization appears less localized. Especially in runs 6–7, the ERD of the left motor imagery is present in both hemispheres, and in the last part of the trial the ERD pattern is similar in the two motor imageries. This leads to lower discrimination between the two classes compared to the PMR and control groups.

**Fig 5:**
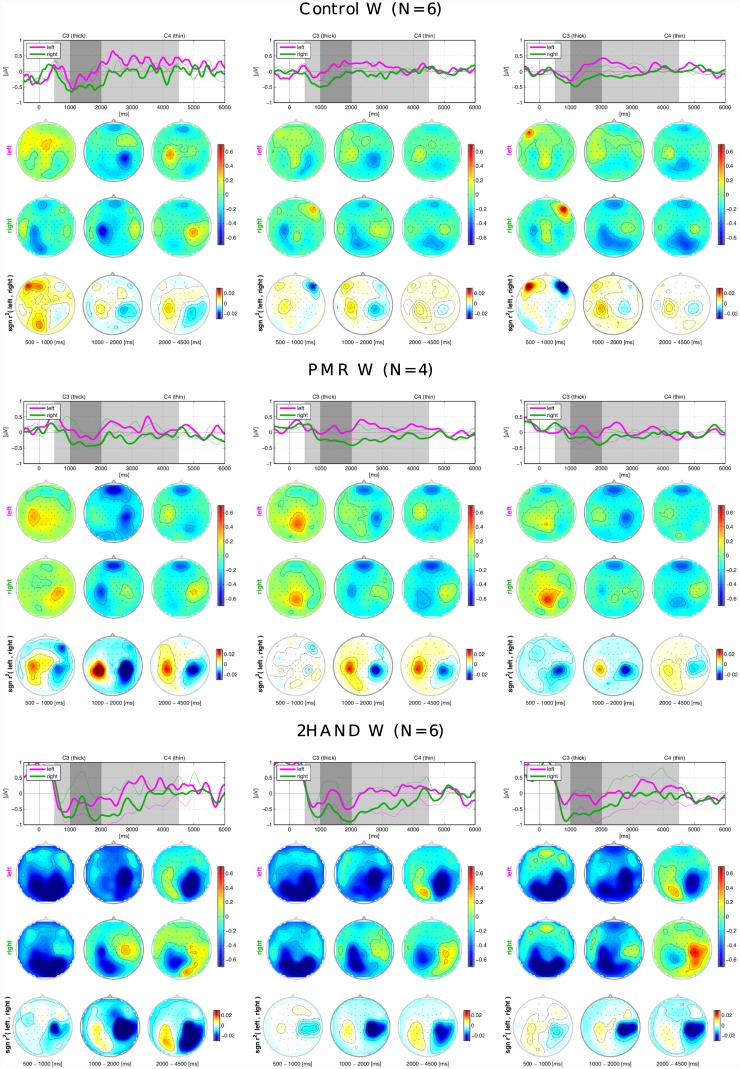
Grand average ERD/ERS for class combination *left-right* and intervention groups in Würzburg. ‘N’ is the number of participants of each group. From left to right: runs 1–3, runs 4–5, runs 6–7. The time plots in the first rows picture the evolution of the ERD/ERS for about 6000 ms at C3 (thick lines) and C4 (thin lines). At time 0 is the onset of the cue, at times 1000–4000 the display of the feedback. Magenta lines refer to *left* MI trials, green lines to *right* MI trials. The scalp plots underneath refer to the shaded areas of the time plots and show the distribution of the ERD/ERS. In the second rows, the scalp plots of the *left* MI trials, in the third rows the scalp plots of the *right* MI trials and in the fourth the scalp plots of the sign-r^2^.

Fig 5 shows the ERD/ERS for the different intervention groups tested in Würzburg, for class combination *left-right*. The PMR group shows clear discrimination between the 2 classes, depicted in the sign-r^2^ scalp plots. We can also notice that in in this group the ERS arises 500 ms before the beginning of the feedback in both classes, in the ipsilateral hemisphere in runs 1–3 and in the central hemisphere in the other runs. The difference between the sign-r^2^ plots of the control and PMR groups is evident and reflects the significant difference between the respective classification accuracies. Besides, in the control group the class-wise discrimination is influenced by muscular artifacts 500 ms before the start of the feedback. The 2HAND group shows great ERD in all the runs of the experiment for both classes. Anyway, analogous to the 2HAND group in Berlin, the ERD are spread through a wider region than the motor cortex. This is visible especially in the scalp plots of the first interval in all the runs. In the intervals 2s-4s instead, the ERD is more focused and also the ERS is visible in the ispilateral motor cortex. The sign-r^2^ scalp plots show clear discrimination between the two classes. Comparing the control groups of Würzburg and Berlin, Control W shows smaller ERD if compared to the Control B and also the discrimination between the two classes is less pronounced. This can be an explanation for the lower classification accuracy of Control W.

### Neurophysiological predictor of BCI performances

**Fig 6:**
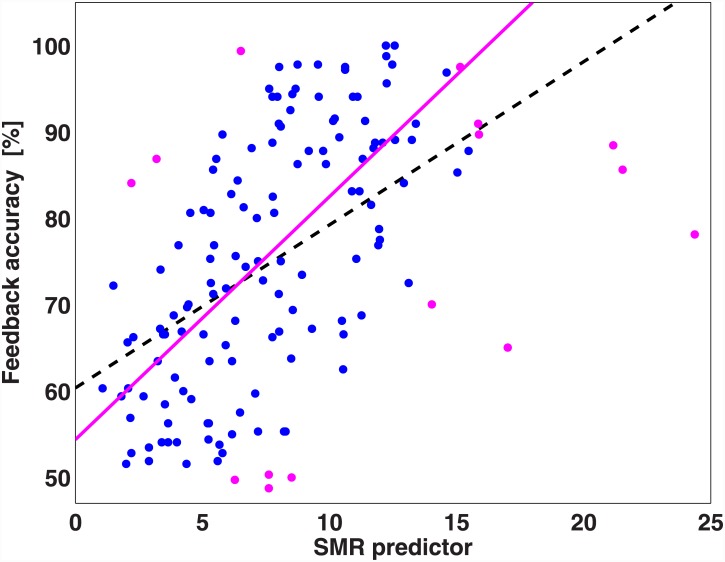
Predictor of BCI performance. The predictor is calculated from 2.5 minutes of recording of the brain in resting state with eyes open and correlated with the mean feedback accuracy for each participant (blue dots). The back dashed line pictures the linear regression between the predictors and the accuracies (r = 0.53). The magenta dots are the values detected as outliers. After the exclusion of the outliers, a higher correlation of 0.66 is reached (magenta line).

The average amplitude of the mu rhythm at rest measured at electrodes C3 and C4 was correlated with the mean BCI performances of each participant. Results are shown in the scatter plot in Fig 6. The values of the predictor significantly correlate with the feedback accuracies (p<0.01), with a Person correlation coefficient of 0.53. Participants having the 10% largest Mahalanobis distances to the data center were considered as outliers [45] and the linear regression was re-calculated. After the outliers’ removal the correlation increased to 0.66. The same method was applied separately to the data of the two laboratories, resulting in a coefficient of 0.55 for participants in Berlin (0.68 after outliers rejection) and of 0.52 for participants in Würzburg (0.60 after outliers rejection). The prediction model derived in [25] was used to estimate the feedback accuracies of the present study. The coefficients of the linear regression were calculated from the previous dataset, after outlier rejection. The estimated accuracies were derived by fitting the linear regression model with the new predictor values extracted by the resting state EEG recording. Pearson linear correlation was then calculated between the estimated accuracies and the real one achieved in the present study, leading to a high significant correlation of r = 0.53 (p<0.01).

## Discussion

The results of our large-scale study run with 151 participants operating a fully co-adaptive SMR-BCI with online feedback since the first trial showed overall mean accuracies of 78.23% in Berlin and 72.44% in Würzburg. In particular, among the 72 data sets considered in these analyses and recorded in Berlin, 50 participants reached mean BCI performances over the threshold of efficient BCI control (>70%), 17 reached mean BCI performances significantly higher than chance level and 5 had performances at chance level. Among the 79 data sets of Würzburg, 40 participants performed over 70%, 28 significantly higher than chance and 11 at chance level. All participants were novel to BCI in general and at their first session with the system. Our findings show that there is still a not-negligible portion of users who did not reach the 70% threshold of BCI performances. These results refer to the average performance of runs 4–7. Reporting the percentage of inefficient BCI users after averaging the accuracies of the whole feedback session is rather pessimistic. In fact, this measure would consider participants having poor performances at the beginning of the session and reaching the 70% at the end as suffering from inefficiency even if they learnt and reached control (e.g. participants in group Control W, Fig 3 (right)). Besides, in previous studies [21, 26] the performance achieved either at the last level or the last run was used to calculate the rate of BCI inefficiency, since participants showed a clear learning trend. Our results instead showed high intra-runs variability for many participants (Fig 2) and the interpretation of the average performances is not straight-forward. Therefore, it is important to distinguish between the percentage of participants whose performance was significantly lower than the control level of 70% (threshold of significance calculated with the binomial test, i.e. 59%) and those who were close to the threshold of control but did not achieve it on average. Using this statistical threshold, the total number of users suffering from inefficiency in the beginning of the session (run 4) was 41 (27%) and decreased to 34 (22.5%) in the end of the session (run 7).

Our previous large-scale study run with 80 participants [27] led to the categorization of BCI users into 3 groups as described in the Introduction and identified some causes of failure in the BCI operation. For some users the problem could be the transition between the offline calibration of the BCI system and its online use with real-time feedback. Other people might not be able to generate discriminative motor imagery patters, therefore leading to the failure of the the system’s calibration. Follow up studies moved a step forward in solving the issue of BCI inefficiency by developing co-adaptive methods in which user and machine mutually learn from each other since the very first trial. For example, in [21] 14 participants were recruited. For 10 participants the separability of brain patterns was not successful in the previous study (Cat III) and 4 participants were novice in respect to BCIs. In one-day session operating a co-adaptive BCI system similar to that described in this paper, five Cat III users reached 70% accuracy in run 2 or 3 and maintained it until the end of the experiment. Two Cat III users started at chance level and significantly improved until the end of the experiment, while the other 3 users performed at chance level. The session was very successful for the 4 novice users, whose performances were always above 70%. The authors concluded that machine learning based co-adaptive calibration was a promising new approach to broaden the applicability of BCI technology. In this study we applied the co-adapative methods to a large sample of BCI novices. The reasons why a not-negligible portion of users did not reach the control level before the end of the session can only be speculated. While it is difficult to explore single-subject reasons for this phenomenon, we can formulate some general hypotheses. One reason can be due to the fact that in the previous studies the selection of the subject specific reactive frequency bands and time intervals after runs 1–3 was done in semi-automatic way, while in our study it was totally automatic. Semi-automatic means that after the automatic selection the BCI expert could visually explore the power spectrum and ERD to check that no wrong parameters were selected. For example it could happen that too narrow time intervals and frequency bands are selected when the classes are not well discernible. Or it might happen that a too late time interval corresponding to beta-rebound is selected [46, 47]. In one of these cases the expert could force the choices of the intervals. While the fully automatic parameters selection would not be a problem for people who are naturally skilled with motor imagery BCIs, it could bring to a sub-optimal choice for those users who do not show clear motor imagery patterns. Another difference can be the frequency of subject-specific parameters update. For example, in [48] the parameters were checked and selected after every run when necessary, while in this study it was only performed after every co-adaptive level. If the update of parameters is not performed regularly enough, the system might lose the chance to identify a change in the parameters that leads to successful performance. Another possible reason could lie in the choice of the positive biased feedback of run 1–3. The feedback given to the user plays an important role in BCI performances. Recent studies demonstrated that visual BCI feedback clearly modulates sensorimotor EEG rhythms [49]. Barber and Wentrup [39] investigated the effect of feedback design on BCI performances, biasing the belief that the participants had on their level of control of the BCI system. They concluded that people who were capable to operate a BCI might be impeded or get frustrated because of inaccurate feedback, while people who performed at chance level may actually benefit form it. In our design, The feedback had two aspects which made it positively biased in runs 1–3: (1) From two binary classifiers that one was chosen (at each time point) which had a better output for the true target. (2) The cursor was never moved from the center towards a wrong location. As a consequence of the way our feedback was biased, it could occur most of the time that the cursor moved in the correct direction just due to chance, in absence of any appropriate activation in the sensorimotor areas. We assume that the classifier was too positively biased and could happen that the person had the feeling to perform correct motor imagery even when that was not the case. Even though participants were aware that the following runs reflected the actual classifier output, the transition between the biased and real feedback could have led to confusion and frustration in some users. Incompetence fear and mastery confidence are key factors to be considered when operating a BCI system, as it was related to BCI performances [50, 51].

We found in our data an unexpected significant effect of the study location on BCI performance, that is Control B performed significantly better than Control W. This is quite surprising, since all the phases of the BCI experiment and psychological interventions were identical (except the duration of 2HAND training) and automatic in the two locations and instruction given in the same way (written form and oral form by BCI experts). The ERD/ERS plots shown in Figs 4 and 5 illustrate that the Control B group already had clear class-wise desynchronization since the first runs. Based on this observation, we speculate that the lab effect might, despite the large number of participant, be just due to chance, i.e. in Berlin a larger number of participants naturally skilled with MI were recruited in the control group. The PMR groups had higher accuracy compared to the control groups, and this difference became statistically significant in Würzburg. This trend reflects the differences in the ERD plots, with the PMR groups having greater desynchronization and better discrimination between classes in respect to control. Regarding the groups performing the 2HAND test we did not find any significant difference in performances compared to the control groups. Anyway, the ERD patterns of these groups differed from the patterns of the other two groups. We could notice a deep desynchronization that appears to be less focused and more spread over the motor cortices. Since the motor coordination training implied the control of the knobs with synchronized coordination of both hands, it caused a simultaneous modulation of the SMR in both motor areas. This effect might temporary influence the following motor imagery and be the reason for the spread ERD patterns.

### Consolidation of the neurophysiological predictor

One goal of this study was to replicate the correlation between the neurophysiological indicator developed by Blankertz et al.[25] and BCI performances on a new large pool of participants. We found a significant positive correlation of 0.53, which raised to 0.66 after outliers rejection, confirming the results of the previous study. Moreover, results show that the proposed model can be transferred between two MI-BCI studies that employ different designs. Indeed, the BCI performances of new participants could be predicted with high significant correlation (p<0.01) by the prediction model derived from the previous dataset [25]. Rarely the replication of a prediction model is reported in BCI literature. An example is described in [52], in which the authors aimed at replicating the two psychological predictors of SMR-BCI performances previously found in [36], in a different BCI setup. They found that the psychological variable ‘visuo-motor coordination ability’ explained a moderate amount of the variance of the SMR feedback performance and could be consolidated as a small predictor of BCI performances. In our study, we found a high significant correlation (p<0.01) between the neurophysiological indicator and BCI performances. The replication of the neurophysiological predictor is a major achievement of this study, since the participants tested were different and also the BCI procedure changed with the introduction of the online feedback since the first trial. These results consolidate the validity of the developed prediction model across different participants and experimental procedures.

### Limitations of the study and future developments

Our findings showed that a relatively large number of users were close to 70% feedback accuracy without reaching it on average over the session. This trend made it difficult to estimate the rates of BCI efficiency. Using a statistical threshold level, the rate of people who could not learn to control the system decreased from 27% to 22.5% in the end of the session. However, this level is arguable low (59%) and the average performance of the session had also to be reported. It is important to note that the comparison with previous studies [21, 26], in which only the final performance was reported, is then not straight-forward. Future developments should pay closer attention to the aspects that showed the weakness of our study. For example, potential users not able to operate successfully the BCI after some runs should get closer assistance from a BCI expert in the calibration of the system and in advising about the motor imagery strategies. Moreover, the positive biased feedback should be revised. For example, we could only bias the feedback not letting the cursor moving towards the wrong direction, which avoids demotivation, while avoiding to choose the best output for the true class, as it more often falsely suggests good performance. (Assuming two random binary classifiers, at least one of them will give the correct output in 75% of the cases on average.) The identification of the users who would potentially need more assistance could be assessed a-priori by the calculation of the neurophysiological predictor. As last suggestion, the proposed methods should be evaluated also with the end users, i.e. patients suffering form serious motor impairments. In fact, the development of new BCI systems must be done always considering that the real beneficiaries of this technology represent a more complex scenario with even higher inter-subjects variabilities.

## Conclusions

Our large scale study conducted with healthy participants showed that about 70% of people could efficiently and consistently operate the fully automatic co-adaptive SMR-BCI system with on-line feedback since the first trial. The reasons for the suboptimal performance of a portion of users were speculated. The differences between previous co-adaptive studies and the system described in this manuscript suggested that the intervention of the BCI expert still plays an important role in the successful performance of SMR-BCIs. The confirmation of the neurophysiological predictor of BCI performance represented an important result that could help in discriminating potential users not able to achieve efficient control with the standard machine learning training of the BCI. For these users different protocols should be adopted or maybe, as the results of the run-wise BCI performances suggested, a longer training would be required to develop an effective modulation of the SMR and more confidence with the system.
